# Aberrant Methylation of LINE-1 Transposable Elements: A Search for Cancer Biomarkers

**DOI:** 10.3390/cells9092017

**Published:** 2020-09-02

**Authors:** Anastasia A. Ponomaryova, Elena Y. Rykova, Polina A. Gervas, Nadezhda V. Cherdyntseva, Ilgar Z. Mamedov, Tatyana L. Azhikina

**Affiliations:** 1 Cancer Research Institute, Tomsk National Research Medical Center, 634009 Tomsk, Russia; anastasia-ponomaryova@rambler.ru (A.A.P.); pgervas@yandex.ru (P.A.G.); nvch@tnimc.ru (N.V.C.); 2 Institute of Chemical Biology and Fundamental Medicine, Siberian Division of the Russian Academy of Sciences, 630090 Novosibirsk, Russia; rykova.elena.2014@gmail.com; 3 Department of Engineering Issues in Ecology, Novosibirsk State Technical University, 630073 Novosibirsk, Russia; 4 Shemyakin-Ovchinnikov Institute of Bioorganic Chemistry, Russian Academy of Sciences, 117997 Moscow, Russia; Imamedov@mx.ibch.ru; 5 Central European Institute of Technology, Masaryk University, 625 00 Brno, Czech Republic; 6 Laboratory of Molecular Pathophysiology, V.I. Kulakov National Medical Research Center for Obsterics, 117997 Moscow, Russia

**Keywords:** LINE-1 (L1), epigenetic cancer biomarker, DNA methylation, cell-free DNA

## Abstract

Cancer remains one of the main causes of human mortality despite significant progress in its diagnostics and therapy achieved in the past decade. Massive hypomethylation of retrotransposons, in particular LINE-1, is considered a hallmark of most malignant transformations as it results in the reactivation of retroelements and subsequent genomic instability. Accumulating data on LINE-1 aberrant methylation in different tumor types indicates its significant role in cancer initiation and progression. However, direct evidence that LINE-1 activation can be used as a cancer biomarker is still limited. The objective of this review was to critically evaluate the published results regarding the diagnostic/prognostic potential of the LINE-1 methylation status in cancer. Our analysis indicates that LINE-1 hypomethylation is a promising candidate biomarker of cancer development, which, however, needs validation in both clinical and laboratory studies to confirm its applicability to different cancer types and/or stages. As LINE-1 is present in multiple cell-free copies in blood, it has advantages over single-copy genes regarding perspectives of using its methylation status as an epigenetic cancer biomarker for cell-free DNA liquid biopsy.

## 1. Introduction

The fifth base, noncanonical 5-methyl-cytosine, was added to the four canonical ones by Rollin Hotchkiss in 1948 [1]. This chemical modification occurs almost exclusively in the context of cytosine base linked to guanosine, termed CpG. Genomes of vertebrates, which are methylated predominantly at CpG sites, are CpG-deficient because 5-methyl-cytosine spontaneously deaminates to thymine [2,3]. The overall CpG-poor genomic landscape is, however, interspersed with loci showing elevated G + C base composition, called CpG islands, which are usually unmethylated in the human genome [3]. There are about 30,000–45,000 CpG islands with an average length of 1–2 kb, which contain 60–70% of G + C compared with 40% of that in the whole human genome [4]. CpG islands are frequently associated with 5′-ends of genes. It has been suggested that approximately 70% of the annotated gene promoters, including those of all house-keeping genes [5], are connected with CpG islands [6]. Strong correlation between CpG islands and the first coding exon [7], and between gene and CpG densities on different chromosomes [4], was noticed for most chromosomes having 5–15 CpG islands per Mb. 

Nowadays, it is widely accepted that DNA methylation has a profound effect on gene expression through transcriptional regulation and is closely involved in numerous cellular processes such as X-chromosome inactivation and imprinting, gene repression, control of cellular development and differentiation, silencing of repetitive elements and maintenance of genome stability [8]. DNA methylation is a key mechanism for repression of transposons—mobile elements which occupy about half of the human genome. Genome rearrangement, which is considered to promote evolution, at the same time could be harmful for individual organisms, and DNA methylation might have been developed as a defense mechanism against transposons as parasites that threaten functional integrity of the genome [9]. 

Epigenetic modification of DNA, in particular methylation of cytosine to 5-methylcytosine, is acknowledged as an important mechanism underlying carcinogenesis [10,11,12]. Aberrant promoter methylation is associated with altered expression of oncogenes and tumor suppressor genes. Hypermethylation of CpG sites in the promoter region of onco-suppressor genes could modify the spatial structure of chromatin, causing transcriptional repression. Different genomic hypomethylation and hypermethylation patterns are characteristic for particular cancer types (the recent meta-analysis is presented in [13]). These patterns could serve as diagnostic and prognostic biomarkers in various analyses, including noninvasive tests such as liquid biopsy [14]. At the same time, global genome hypomethylation observed in repetitive sequences and intergenic regions, is one of the hallmarks of cancer cells [15], and is suggested to promote oncogenesis by increasing the mobility of transposons, that leads to genomic instability [16,17,18,19,20]. 

## 2. LINE-1 Retrotransposons in the Human Genome

Repetitive sequences constitute approximately half of the human genome and are subdivided into two groups: satellites, or tandem repeats, which represent repeated sequences of one or more nucleotides adjacent to each other, and interspersed repeats, which are scattered throughout the genome [20]. The interspersed repeats are derived from transposable elements of two major types—DNA transposons and retrotransposons. In turn, the latter are divided into those with and without long terminal repeats (LTR and nonLTR retrotransposons, respectively) [21]. Most transpositional activity in the human genome is executed by nonLTR retrotransposons, particularly long interspersed elements (LINEs), among which LINE-1 (or L1) makes up about one-sixth of the genome. L1s are amplified to more than 500,000 copies [4]. However, most of them are rendered inactive by 5′-truncations producing L1 elements of ~0.9 kb in average. Only about 80–100 L1 copies are full length (∼6 kb) and contain two open reading frames (ORF1 and ORF2) flanked by 5′- and 3′-untranslated regions (UTRs). ORF1 encodes an RNA-binding protein (ORF1p) and ORF2—a protein with endonuclease and reverse-transcriptase activities (ORF2p) [20], which are responsible for L1 retrotransposition through the process termed target primed reverse transcription [22]. In its 5′-UTR, L1 has an internal promoter for RNA polymerase II, mapped to the +1–909-bp region, which also contains an antisense promoter for transcription of the third open reading frame (ORF0) with unknown function [23]. The first 460-bp portion of the 5′-UTR includes 29 CpG sites, which are heavily methylated in normal somatic cells [8,24].

As only a small number of L1s are full length and potentially active [25,26], these elements are considered fossils from the genetic past and are often referred to as junk or parasitic DNA. However, accumulating evidence suggests that L1s play an important role in various cellular processes, including carcinogenesis. Although in many cases L1 transpositional activity or hypomethylation seem to be a part of global cellular dysregulation associated with carcinogenesis [27], L1s can alter gene expression or modify genomic structure in different ways. In particular, L1 insertions into exons or introns can disrupt gene sequence and abolish gene expression [28,29,30,31]. Alternatively, insertions into the regulatory region can activate gene expression leading to malignant transformation. For example, L1 insertion into enhancer of suppression of tumorigenicity 18 (ST18) gene disrupts a negative feedback loop and can play a role in the development of hepatocellular carcinoma [31]. It was also shown that homologous recombination between two L1 copies with similar sequences could result in chromosomal rearrangements, including large inversions, duplications or chromosome fusions [32,33], which, in turn, can affect gene expression. Recent investigation of nearly 3000 individual genomes from 38 histologically different cancer subtypes revealed multiple examples of megabase-scale chromosomal deletions induced by L1 [34]. Such deletions can occasionally remove tumor-suppressor genes (e.g., *CDKN2A*) or trigger the amplification of oncogenes (e.g., *CCND1*). All these L1 activities can participate in the pathogenesis of many diseases, including cancer [35,36,37,38]. Thus, it was shown that in cancer cells, L1 retrotransposons could inactivate gene function through insertional mutagenesis, aberrant splicing or DNA breaks, leading to genomic instability [39,40]. In tumor cells, somatic L1 insertions occur more often in intergenic or heterochromatic regions [41], in cancer-specific hypomethylation areas [42] and in genes commonly mutated in cancer, indicating an oncogenic nature of L1 transductions [39,42,43].

### 2.1. L1 Methylation in Cancer Diagnostics

**Figure 1:**
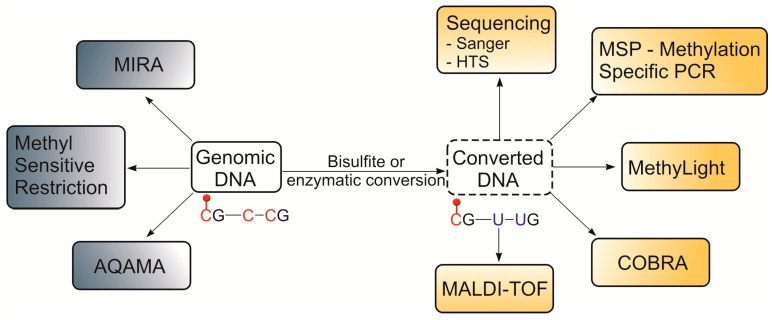
Methods used for L1 methylation analysis in cancer. For further details, see: MSP (methylation specific PCR, [47]), COBRA (combined bisulfite restriction analysis, [48]), MethyLight ([49]), MALDI TOF (matrix-assisted laser desorption ionization–time of flight, [50]), MIRA (methylated-CpG island recovery assay, [51]), and AQAMA (absolute quantitative assessment of methylated alleles, [52]).

The L1 methylation status is usually assessed by techniques based on chemical or enzymatic conversion of cytosine residues to uracil (without affecting 5-methyl-cytosine) followed by sequence analysis. Alternative methods can discriminate between methylated and unmethylated cytosines directly without conversion. Figure 1 presents current approaches most widely used for L1 methylation analysis, which are described in detail in several comprehensive reviews [44,45,46]. Quantitative assessment of the L1 methylation level is performed based on the methylation index (MI) defined as the ratio of methylated to the total number of CpG sites within a locus.

The diagnostic and prognostic significance of L1 methylation in cancer is summarized in Table 1. L1 hypomethylation is proposed to be an initiating factor in carcinogenesis because, in some cancers, significant demethylation is observed in premalignant lesions before cancer transformation. Barchitta et al. [53] conducted meta-analysis to determine the diagnostic value of tissue L1 hypomethylation in cancer. The authors selected 19 unique articles describing 20 different cancer types and several methods to detect L1 methylation; a total of 2554 cancer samples (1127 tissue and 1427 blood samples) and 3553 control samples (2811 from healthy subjects and 742 from tumor-adjacent normal tissues) were analyzed. The results indicated that the overall L1 methylation level was 6.4% lower in cancer tissues than in normal tissues. However, the inter-study heterogeneity was high and the difference in methylation was confirmed significant for tissue but not for blood samples [53].

Colorectal cancer (CRC) is one of the most studied cancer types regarding the L1 hypomethylation status. L1 hypomethylation assessed by absolute quantitative analysis of methylated alleles (AQAMA) was shown to be an early event in colon cancer progression. L1 MI was reported to be significantly lower in adenoma samples than in normal colon mucosa and was suggested as a biomarker to differentiate between adenoma and normal tissue [72]. There were significant differences in the L1 methylation level depending on CRC stage and prevalence, but no such differences were observed between adenoma and carcinoma [73], indicating that L1 hypomethylation may potentially serve as a biomarker for early-stage CRC before its transition from premalignant to malignant lesions. Detection of precancerous adenoma formation is of great importance as it provides an opportunity for timely surgical intervention in CRC, thus reducing the risk of fatal colon adenocarcinoma development and improving patient survival [73] (Table 1).

L1 hypomethylation is also an early event in breast tissue transformation and has been observed in atypical ductal hyperplasia and flat epithelial atypia. Similar to colon cancer, L1 methylation was shown not to decrease significantly in the course of progression from the hyperplastic to breast carcinoma state [55]. However, another study indicated that methylation levels in normal tissues and tissues with atypical ductal hyperplasia were comparable, and significant changes were detected only in ductal carcinoma in situ and AJCC (American Joint Committee on Cancer) stage I breast cancer [54]. Nevertheless, both studies reveal that the L1 methylation status is associated with breast cancer, suggesting its potential as a companion diagnostic marker.

Esophageal cancer is among the top 10 most fatal cancers, with a 5-year survival rate of 43% [74]. Early diagnosis of esophageal cancer improves 5-year survival, highlighting the importance of establishing a reliable diagnostic biomarker. Unfortunately, the sensitivity of endoscopy screening for esophageal cancer is poor, as up to 40% patients with surgically proven invasive disease showed negative endoscopy results [74]. Currently, there are no good diagnostic markers for esophageal cancer, and proposed indicators such as expression of proinflammatory COX2 and NF-κB do not show sufficient specificity [74]. Several studies have reported L1 hypomethylation in esophageal squamous cell carcinoma (ESCC). Shigaki et al. [59] revealed significant correlation between smoking history and hypomethylation in noncancerous esophageal mucosa of 109 patients with ESCC, suggesting possible involvement of L1 in the pathogenesis of esophageal cancer due to environmental factors. By using combined bisulfite restriction analysis (COBRA), Chalitchagorn et al. [75] found that in ESCC, the L1 hypomethylation level ranged from 23 to 50%, which is on average 10% lower than that in normal esophageal tissue. Zhu et al. [57] also detected L1 hypomethylation in 310 ESCC cases using real-time methylation-specific PCR, further confirming a significant decrease of L1 methylation in esophageal cancer compared to nontumor tissue (Table 1).

L1 methylation status has also been studied in prostate and ovarian cancers, albeit to a lesser extent than for the cancer types discussed above. Using methylation-sensitive restriction enzyme digestion of tissue-isolated DNA and an L1-specific ^32^P-labeled probe, Schulz et al. [76] found that 31% (17/55) of prostate carcinomas had more than a 10% decrease in L1 methylation compared to normal tissue, which was correlated with chromosome 8 instability, indicating the diagnostic potential of a combination of epigenetic and genetic markers in prostate cancer. The hypomethylated status of L1 in prostate cancer has been further confirmed by Santourlidis et al. [77], who observed it in 53% (17/32) of prostate cancer samples analyzed by semiquantitative PCR. L1 hypomethylation has also been reported as an early event in the initiation of ovarian cancer. Thus, Pattamadilok et al. [78] used COBRA to assess L1 methylation and showed that it was lower in epithelial ovarian cancer tissues than in normal ovarian tissues (34.9% vs. 46.9%; *p* < 0.001).

However, to date there is no unified opinion on the clinical significance of the L1 methylation status in cancer diagnostics, which could be attributed to the lack of a standard approach to estimate L1 methylation, as well as to differences in sample size and homogeneity among the studies. Adequate representation of patient subgroups according to clinical and morphological characteristics and universal detection protocols are necessary requirements to conduct a validation study in order to confirm the reliability of L1 methylation as a cancer diagnostic biomarker.

### 2.2. L1 Methylation in Cancer Prognosis

Hypomethylation of L1 has also been shown to correlate with prognosis for many cancer types (Table 1). Thus, CRC prognosis has been significantly associated with L1 hypomethylation as well as with the global methylation status [62,63,64,79]. Ogino et al. [79] examined the levels of L1 methylation in 643 colon cancer samples at different disease stages using pyrosequencing, and found that L1 hypomethylation was linearly associated with poorer prognosis: a 30% decrease in L1 methylation corresponded to colon cancer-specific mortality (hazard ratio [HR] 2.37) and overall mortality (HR 1.85). The same correlation was reported in a more recent study of Kaneko et al. [64], who evaluated L1 methylation in metastatic and recurrent tumors from 40 patients with CRC using a MethyLight assay. In line with these observations, Swets et al. [65] reported a significant correlation between L1 hypomethylation and overall survival of patients with colon cancer. However, unlike colon cancer, rectal cancer showed low rates of microsatellite instability-driven tumors and, therefore, was suggested to be less prone to L1 activation as an initiating event in tumorigenesis.

The risk of developing aggressive hepatocellular carcinoma has also been linked to L1 hypomethylation. Zhu et al. [57] used pyrosequencing to demonstrate that hypomethylation of three specific CpG sites in the L1 5′-UTR was strongly associated with poor prognosis, advanced stage, and the risk of metastasis and vascular invasion. They also found that activation of the proto-oncogene c-Met, a high-affinity receptor for hepatocyte growth factor and a critical element in the growth and metastasis of hepatic tumors, was associated with hypomethylation of L1 inserted upstream of the MET gene. Similarly, Gao et al. [56] reported correlation between poor prognosis in patients with hepatocellular carcinoma and hypomethylation of another two L1 5′-UTR sites analyzed by using bisulfite-specific PCR and DNA sequencing.

The association of L1 hypomethylation with poor prognosis has been observed for esophageal, breast, bladder and lung cancers [54,55,59,60,68,80]. Thus, it was found that decreased L1 methylation was indicative of early progression from metaplasia to adenoma in gastric cancer, where methylation levels < 51% were significantly correlated with decreased overall and disease-free survival [62]. L1 hypomethylation in breast cancer was reported to indicate disease progression [55], particularly for primary tumors [54]. In a study of 310 patients with ESCC, lower L1 methylation was detected in cancer compared to normal tissues and was associated with poorer prognosis: for patients with a total MI of <78%, mean overall survival was 34 months, whereas for those with an MI of >78% it was 43 months [61].

Although these data support the hypothesis that L1 hypomethylation is a prognostic marker in cancer, contradictory results have been reported in the literature, which could be due to a low number of patients examined. Another important reason to interpret the data on L1 methylation with caution is that in many cancers, hypomethylation of L1 may serve as a surrogate indicator of activation of other cancer-related genes involved in tumorigenesis, which represents a major disadvantage of using L1 hypomethylation as a biomarker in cancer prognosis, as well as diagnosis.

## 3. Circulating DNA as a Source of Cancer Biomarkers

Biopsy tissue samples are a standard source of material for tumor molecular analysis used to confirm diagnosis, detect cancer-specific mutations and classify tumor types, as well as to guide therapeutic interventions [81]. However, tissue biopsy is an invasive and often challenging procedure. Furthermore, it sometimes does not provide enough material to perform several molecular tests required for making a reliable diagnosis. Another issue is spatial and temporal tumor heterogeneity, which decreases the practical utility of biopsy as a tool to monitor cancer progression and evaluate response to therapy [82].

Tumors shed multiple components into the bloodstream that travel throughout the body, including cancer cells and their DNA, RNA, proteins and exosomes, which can be used as blood-circulating tumor biomarkers that would provide similar information to tissue biopsy, pinpoint the primary site of cancer origin, and be instrumental in routine monitoring of cancer progression or therapeutic efficacy [83]. Furthermore, liquid biopsies are less invasive and expensive, and can be collected at different time points during the course of treatment [82,84].

Circulating cell-free DNA (cfDNA) isolated from blood of cancer patients contains fragments shed by tumor cells, which can be used to assess the molecular profile of cancer [85,86]. Recently, the cell surface-bound fraction of circulating DNA (csb-cirDNA) has been proposed as an additional source of cfDNA [87]. Tumor cells shed mutated DNA, also known as tumor cirDNA, which is now regarded as a highly specific prognostic marker for some cancers [88].

Analysis of aberrantly methylated sequences in circulating cfDNA is one of the most promising approaches for establishing a convenient and effective diagnostic system [89]. Aberrantly methylated cfDNA fragments can be detected in blood by PCR, regardless of tumor localization, and have been reported for all tumor types and development stages [90,91]. However, despite all the benefits, only one cancer targeted diagnostic system, the Epi proColon test (Epigenomics AG, Berlin, Germany), which is based on the detection of a methylated Septin 9 gene in cfDNA, has received approval for commercial use from the FDA. Another FDA-approved product from the same company, Epi proLung test, is based on the detection of methylated *SHOX2* and *PTGER4* genes in bronchial flushes, where DNA at least partially originates from lung tissues. This indicates that a number of challenges should be overcome for the novel cfDNA-based tests development and implementation into clinical practice.

Currently, the principal targets of blood cfDNA analysis are hypermethylated tumor suppressor genes. However, they are usually single-copied, which makes it challenging to detect their methylated alleles contained in the bloodstream in very low amounts (typically a few nanograms per milliliter) [92,93]. Therefore, analysis of the methylation status of mobile genomic elements, in particular L1s, which are abundantly present in the human genome, is a promising approach aimed to improve sensitivity of the methylation-specific based test [94].

### 3.1. Methylation of Circulating L1 in the Healthy State

It is considered that hypomethylation (demethylation) and related chromosomal instability are associated with age and age-related pathologies. However, studies that investigated the link between DNA methylation and aging did not produce conclusive results. Thus, El-Maarri et al. [95] showed that aging did not affect L1 methylation in peripheral blood cells, whereas Bollati et al. [96] found weak association of L1 methylation with aging in blood cells. However, the cohorts of the two studies significantly differed in age (18–64 years vs. 55–92 years, respectively), which may account for inconsistent results. Another factor may be the use of blood cell DNA for L1 methylation analysis. In blood, cellular DNA originates from short-living leukocytes (neutrophils, lymphocytes, monocytes, eosinophils and basophiles) [95], which show stable methylation of repetitive DNA elements in individuals of 20–61 years old in whom these cell populations are rapidly renewed [97]. In contrast to blood cells, neuronal cells accumulate changes in L1 methylation in course of aging. L1 activity caused by derepression, including hypomethylation, has been found to be characteristic for the aging brain and to be associated with age-dependent neurological disorders [98]. In blood, cfDNA rather than cellular DNA could be a better object for methylation analysis because cfDNA is released from apoptotic and necrotic cells and, as such, represents a more reliable source of biomarkers for aging. Indeed, it has been shown that L1 hypomethylation in cfDNA, but not cellular DNA, from peripheral blood is associated with human age [95,97].

Therefore, it could be suggested that genome-wide hypomethylation occurs in an age-dependent fashion in many types of cells from various organs, which could lead to retrotransposon activation, induction of genomic instability [99], aberrant transcription patterns [100] and increase in cell apoptosis [101]. On the other hand, the opposite process, hypermethylation of certain promoter regions including those of tumor suppressor genes, can occur during aging and result in the development of various pathologies [102].

Methylation of retrotransposable elements can also be deregulated by lifestyle factors such as smoking. It was shown that L1 hypomethylation in esophageal and oral mucosae was significantly associated with tobacco use [59,103] and was increased in respiratory epithelium exposed to cigarette smoke [104]. Harmful components of smoke, e.g., nicotine, induce apoptosis in various types of cells [105], which can release hypomethylated cfDNA into circulation as evidenced by detection of hypomethylated L1 and *Alu* retrotransposable elements in cfDNA of smokers [97]. These findings suggest that the degree of cfDNA demethylation could be a surrogate indicator of smoking-induced adverse effects on human health, which may be used for monitoring health improvement after quitting smoking. It should be noted that using the cfDNA methylation status alone as a marker is not sufficient to identify the damaged tissue, but it can generally mirror the pathological or age-associated changes in the organism.

Thus, L1 hypomethylation in cfDNA can be used as one of the nonspecific indicators of human age and/or health status.

### 3.2. Perspectives of Using Aberrantly Methylated Circulating L1 for Cancer Diagnostics and Prognosis

**Figure 2:**
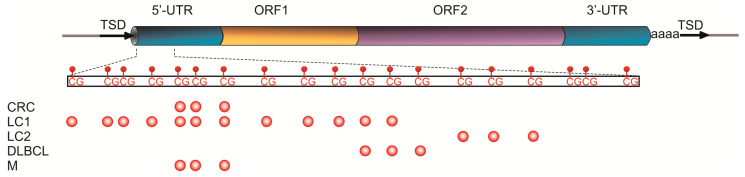
Hypomethylation of the L1 promoter region in cfDNA of different cancer types. Top, schematic structure of the L1 retroelement: TSD, target site duplication; 5′-UTR and 3′-UTR, 5′- and 3′-untranslated regions, respectively; ORF1 and ORF2, open reading frames 1 and 2, respectively; the magnified region shows distribution of CpG sites in the L1 promoter. Bottom, CpG sites (red circles) in the L1 promoter evaluated in different cancer types: CRC, colorectal cancer [112]; LC1 [115] and LC2 [116,117,118], lung cancer; DLBCL, diffuse large B cell lymphoma [119]; M, melanoma [120].

In CRC diagnosis, colonoscopy is the gold standard showing over 90% sensitivity and specificity [106]. However, most patients are reluctant to undergo the procedure because of its invasive nature and possible morbid complications such as bowel perforation. The fecal occult blood test (FOBT) is the most frequently used noninvasive diagnostic modality in CRC screening, but it has serious limitations such as relatively low sensitivity for early-stage or proximal colon cancer [107,108]. Moreover, the adherence rate to the CRC screening program based on FOBT and colonoscopy remains low (about 50%) [109,110], and it is reported that many people who avoid FOBT prefer a simple blood-based test instead [111]. Currently, the most frequently used blood biomarker for CRC screening is carcinoembryonic antigen (CEA), although it is considered to be insufficient for reliable CRC diagnosis. In a recent study, Nagai et al. [112] suggested using the L1 MI measured by AQAMA in cfDNA (see Figure 2 and Table 2 for details) as an alternative biomarker for CRC blood-based detection. The authors found that L1 MI was significantly higher in patients with both early (I/II) and advanced (III/IV) CRC stages compared to healthy donors, and showed that L1 MI surpassed CEA in the detection of early-stage CRC and had a similar sensitivity for advanced-stage CRC. It should be mentioned that there was no difference in the MI of L1 between CRC stages, which is in contrast with the results obtained in tissue samples, indicating that L1 hypomethylation occurs at a very early stage of CRC development [72]. However, several studies have reported that the L1 hypomethylation status in CRC tissues is independent of cancer stage [62,79,113,114] and is believed to remain relatively stable during CRC progression [35], which may partially explain the absence of difference in L1 methylation between cfDNA from patients with early-stage and advanced CRC. On the other hand, patients with CRC of a very advanced stage (N ≥ 2 and M1), and with a large tumor size (≥6.0 cm), showed significantly decreased L1 MI in cfDNA compared to other patients. These results suggest a principal possibility of using methylation of cfDNA-derived L1 as an additional noninvasive biomarker which, in combination with other biomarkers such as FOBT and CEA, can indicate an increase or a decrease of likelihood for advanced CRC and/or distant metastasis.

Global hypomethylation of L1 in cfDNA has been linked to disease progression in several cancers. Wedge et al. [119] assessed the prognostic value of L1 methylation in diffuse large B cell lymphoma by pyrosequencing of cellular DNA from 67 tumor biopsies and plasma cfDNA from 74 patients. The results support the concept of diagnostic utility of liquid biopsy, as they indicated that global hypomethylation in cfDNA was a strong prognostic factor correlating with overall survival. Potential limitation of the approach was also noted, as two of the nine patients for whom tumor and blood samples were tested in parallel did not show hypomethylation in cfDNA. In one of them, the discrepancy could be attributed to an early disease stage, when less tumor DNA is observed in circulation [88]. Because of a small number of patients (*n* = 9) with both tumor and liquid biopsies in this study, the sensitivity of cfDNA testing for global L1 hypomethylation could not be evaluated. Further investigation in a large cohort of patients with matching tumor and blood biopsies is required.

In lung cancer, studies on L1 methylation in cfDNA are limited. In our recent work, we revealed a significant difference in L1 promoter methylation of cell surface-bound csb-cirDNA between patients with lung cancer and healthy donors (Table 2). L1 methylation was analyzed in patient blood before treatment and at several stages of antitumor therapy (*n* = 16) using methylation-specific qPCR [117]. It was found that the dynamics of L1 methylation depended on the tumor histological type. Thus, in squamous cell carcinoma, changes in the L1 MI were more pronounced after the completion of treatment, whereas in adenocarcinoma, the most substantial change occurred at the first stage of treatment (after chemotherapy). Importantly, changes in the L1 MI of csb-cirDNA depended on the patient response to chemotherapy, indicating a close association between this serological marker and the pathological process in lung cancer.

In another study, we used the methylated CpG island recovery assay (MIRA) to compare integral methylation in L1 promoters of csb-cirDNA from patients with lung cancer (*n* = 59) and healthy controls (*n* = 47) [115] (Table 2). The results revealed a significant difference in L1 promoter methylation between cancer patients and healthy individuals; furthermore, hypomethylation was more pronounced for the human-specific L1Hs family.

Comparison of L1 MI in patients with lung cancer (*n* = 23) and a joint control group comprising healthy individuals and patients with bronchitis and chronic obstructive pulmonary disease (COPD) (*n* = 47) showed a statistically significant decrease of L1 methylation in cancer samples [118]. There was also a decreasing tendency for L1 MI in patients with lung cancer versus those with COPD (Mann-Whitney U-test, *p* = 0.07). These data indicate that quantitative analysis of L1 methylation in csb-cirDNA is valuable for discrimination of patients with lung cancer from those with chronic inflammatory lung diseases.

Hoshimoto et al. [120] showed that serum levels of unmethylated L1 were higher in patients with stage III-IV melanoma compared to healthy donors (Table 2) and suggested a significant diagnostic potential of the L1 hypomethylation status in cfDNA in melanoma.

Figure 2 summarizes CpG sites investigated for methylation in the studies cited in Table 2. Although these studies used different methods to target distinct CpGs within the L1 promoter region, all of them demonstrated L1 hypomethylation in cfDNA in different cancer types. Summarizing these results, one can assume that analysis of L1 methylation status in cfDNA could be either a universal cancer marker or a marker of a number of cancer types. One of the limitations of cfDNA-based markers is the inability to distinguish the damaged tissue which cfDNA originates from. However, cfDNA can mirror the state of the body in general. Additional studies of L1 methylation status in cfDNA from patients with different cancers and nonmalignant disorders are necessary to evaluate its significance and reliability for clinical use. In addition, any novel blood-based cancer marker should be used with caution and in combination with other biomarkers. 

## 4. Final Remarks

Cancer is one of the main causes of human mortality despite the significant therapeutic progress achieved in the past decade. CfDNA from blood plasma is an established marker of processes characteristic for malignant cells and their microenvironment [121,122,123]. Therefore, the use of cfDNA (liquid biopsy) for early cancer detection, therapy monitoring and relapse prediction is under intensive investigation as a noninvasive method that, in particular, can detect localization of metastases. However, the potential of liquid biopsy is limited by the low concentration of tumor DNA in the circulation, which is translated into insufficient sensitivity and complicated wet-lab and computational procedures. As a result, the only cfDNA-based test for colon cancer diagnostics currently approved by the FDA for clinical use is Epi proColon (Epigenomics AG, Germany).

**Figure 3:**
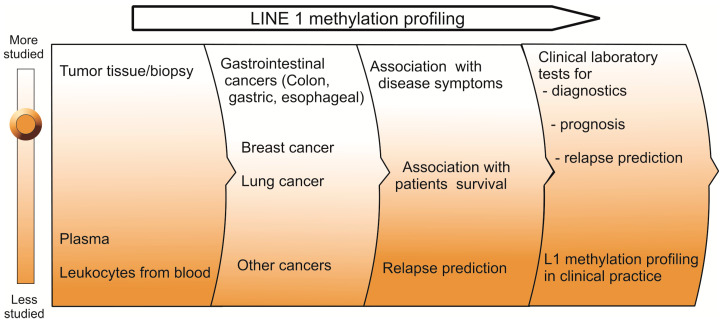
Direction of research on L1 methylation as a road to establish routine noninvasive testing of cancer patients in clinics. Brown gradient corresponds to the volume of knowledge at every stage.

Massive hypomethylation of retroelements, in particular L1s, is considered a universal hallmark accompanying many malignant transformations, including gastrointestinal, breast and lung cancers (Figure 3). L1 methylation levels in cancer have been extensively studied as potential epigenetic markers for diagnostic and prognostic purposes. In 2019 the Pangea Laboratory issued the Bladder CARE™ test, which is a urine assay for bladder cancer detection based on the combination of hyper- and hypomethylation of a three-gene panel, *SOX1*, *IRAK3*, and L1 [124]. Analysis of L1 hypomethylation in cfDNA is a very promising approach to circumvent the limitations of the liquid biopsy mentioned above, because cancer cells can release multiple L1 copies so that the concentration of transposon DNA in blood is significantly higher than that of single-copy genes. However, L1 methylation in cfDNA is poorly studied compared to that in tumor tissues, and has been described for only four cancer types [112,117,119,120]. Although these pioneering works showed very interesting results, further efforts are needed to address critical limitations of research related to L1 hypomethylation in cfDNA in order to determine its applicability as a biomarker in clinical practice. One of the significant limitations of cfDNA-based biomarkers is that the tissue of the damaged cfDNA origin stays enigmatic. Therefore, L1 methylation in cfDNA should be used as an additional marker indicating the increased probability of a malignant disease. Further studies on larger cohorts of patients with different tumor types and stages should be conducted to reduce the level of inter-patient heterogeneity; furthermore, a broader range of CpG sites should be tested and different cancer types analyzed.

The identification of L1 hypomethylation patterns characteristic for each tumor category is of particular interest. Different tumors are characterized by distinct methylation patterns, which inevitably should be reflected in L1 methylation profiles. Such studies are methodologically challenging: L1 copies have very similar sequences and it is hard to distinguish them by currently available methods, including high-throughput sequencing. Identification of individual copies and unique genomic regions adjacent to each L1 element can be used in combination with unique molecular identifiers [125] that allow matching of converted sequences with their nonconverted templates. Advanced computational approaches such as machine learning should also be employed to determine cancer type-specific L1 hypomethylation signatures in cfDNA. Clinical implementation of cfDNA-derived L1 methylation profiling in cancer could address the challenges of highly sensitive screening for early-stage tumor detection, independently of its location, and identification of a specific tissue undergoing malignant transformation.

## Figures and Tables

**Table 1:** Diagnostic and prognostic significance of the L1 methylation status in cancer.

Cancer Type	Methods	Diagnostic Value (*)	Prognostic Value	Ref.
Breast cancer	Pyrosequencing	Normal tissues—92%, IBC tissues—86%	L1 hypomethylation was significantly associated with decreased OS (HR 2.19, 95% CI 1.17–4.09), decreased DFS (HR 2.05, 95% CI 1.14–3.67), and increased DR (HR 2.83, 95 % CI 1.53–5.21) in younger (≤55 years) but not in older patients (>55 years)	[54]
	Pyrosequencing	Normal tissues—64%, IBC tissues—61%	-	[55]
Hepatocellular carcinoma	Bisulfite-specific PCR and DNA sequencing analysis	Normal tissues—60%, tumor tissues—34%	Patients with L1 hypomethylation had decreased median postresection TFS (22 months [95% CI: 13.3–30.7]) and OS (35 months [95% CI: 24.0–46.1]) compared to those with L1 hypermethylation (40 and 60 months, respectively)	[56]
	Pyrosequencing	Normal tissues—68%, tumor tissues—48%	-	[57]
		Normal tissues—57%, tumor tissues—46%	-	[58]
Esophageal cell carcinoma	Pyrosequencing	Normal tissues—82%, tumor tissues—64%	-	[59]
		Normal tissues—79%, tumor tissues—63%	L1 methylation was significantly associated with DFS (univariate HR 2.32, 95% CI 1.38–3.84, methylation level [quartile] < 56%; multivariate HR 1.81, 95% CI 1.06–3.05) and CSS (univariate HR 2.21, 95% CI 1.33–3.60; multivariate HR 1.87, 95% CI 1.12–3.08)	[60]
	Quantitative real-time MSP	Normal tissues—90%, tumor tissues—78%	Cumulative survival was significantly shorter for ESCC patients with L1 methylation level ≤ 78% than for those with > 78% (34 vs. 43 months)	[61]
Colorectal cancer	Pyrosequencing	Normal tissues—77%, tumor tissues—57%	OS was significantly longer in patients with L1 methylation level ≥ 65%	[62]
	MSP-PCR, pyrosequencing after assay validation	-	L1 hypomethylation was significantly associated with higher CRC-specific mortality (for 10% decrease in L1 methylation: HR 2.45, 95% CI 1.64–3.66)	[63]
	MethyLight assay	-	PFS, OS, and 5-year OPS were significantly shorter in patients with low L1 methylation than in those with high L1 methylation (HR 1.00 vs. HR 2.74 [95% CI 1.19–6.29])	[64]
	Quantitative PCR	-	L1 hypomethylation was significantly associated with lower OS (HR 1.68, 95% CI 1.03–2.75); the association was stronger in patients > 65 years (HR 2.00, 95% CI 1.13–3.52)	[65]
Gastric and colon cancers	Pyrosequencing	Colon: normal tissues—67%, tumor tissues—61% Gastric: normal tissues—66%, tumor tissues—62%	-	[66]
Gastric cancer	Pyrosequencing	Chronic gastritis—62%, cancer—52%	L1 hypomethylation level (<51%) was significantly associated with shorter DFS and OS	[67]
Lung cancer	Pyrosequencing	Normal tissues—74%, ADC tissues—67%	Patients with low L1 methylation levels (19–69%) had significantly higher recurrence rates and shorter DFS compared to those with high methylation levels (74–81%)	[68]
		-	L1 hypomethylation (<52%) was significantly associated with lower survival rates in patients with ADC stage I	[69]
	Bisulfite-PCR, pyrosequencing	Normal tissues—70%, ADC tissues—63%, SCC tissues—38%	L1 hypomethylation (≤58%) was independently associated with poor prognosis (*p* = 0.025)	[70]
Oropharyngeal squamous cell carcinoma	Quantitative MSP-PCR	-	L1 hypomethylation (<50% vs. ≥70%) was significantly associated with higher risk of early disease relapse (OR = 3.51; 95% CI 1.03–12.00)	[71]

Abbreviations: IBC, invasive breast cancer; CRC, colorectal cancer; ESCC, esophageal squamous cell carcinoma; OS, overall survival; DFS, disease-free survival; DR, distant recurrence; TFS, tumor-free survival; OPS, overall probability of survival; CSS, cancer-specific survival; PFS, progression-free survival; SCC, squamous cell carcinoma; ADC, adenocarcinoma. *—Diagnostic value is considered according to L1 MI.

**Table 2:** Methylation of circulating L1 in blood samples of cancer patients.

Tumor Location	Clinical Samples	Method	Results	Ref.
Colorectal (patients before treatment)	Plasma	AQAMA qPCR	Significant decrease of L1 MI in cancer patients compared with healthy subjects Association of L1MI with disease progression (advanced stage and distant metastasis)	[112]
Lung (patients before treatment)	Cell surface-bound fraction of blood	MIRA	Significant decrease of L1 MI in cancer patients compared with healthy subjects Hypomethylation of L1 promoters in cancer patients is more pronounced for the L1 human-specific (L1Hs) family	[115]
Lung (patients before treatment and after antitumor therapy)	Cell surface-bound fraction of blood	qMSP PCR	Association of L1 MI with tumor histological type Dynamic changes in L1 MI of csb-cirDNA during the follow-up period	[117]
Lung (patients before treatment)	Cell surface-bound fraction of blood, plasma	qMSP PCR	Significant decrease of L1 MI in csb-cirDNA in cancer patients compared with healthy subjects	[116]
Lung (patients before treatment)	Cell surface-bound fraction of blood, plasma	qMSP PCR	Decrease of L1 MI in cancer patients compared with the joint control group (healthy subjects + patients with bronchitis + COPD patients) and with COPD patients	[118]
Diffuse large B cell lymphoma (patients before treatment)	Plasma	Pyrosequencing	Association of L1 hypomethylation with poor overall survival	[119]
Melanoma (patients before treatment)	Serum	AQAMA qPCR	Decrease of L1 methylation during disease progression (advanced stage)	[120]

Abbreviations: MIRA, methylated CpG island recovery assay; AQAMA, absolute quantitative assessment of methylated alleles; qMSP-PCR, quantitative methyl-specific PCR; L1 MI, L1 methylation index; csb-cirDNA, cell surface-bound circulating DNA.

## References

[1] Hotchkiss R.D. (1948). The quantitative separation of purines, pyrimidines, and nucleosides by paper chromatography. J. Biol. Chem..

[2] Bird A.P. (1980). DNA methylation and the frequency of CpG in animal DNA. Nucleic Acids Res..

[3] Deaton A.M., Bird A. (2011). CpG islands and the regulation of transcription. Genes Dev..

[4] Lander E.S., Linton L.M., Birren B., Nusbaum C., Zody M.C., Baldwin J., Devon K., Dewar K., Doyle M., FitzHugh W. (2001). Initial sequencing and analysis of the human genome. Nature.

[5] Zhu J., He F., Hu S., Yu J. (2008). On the nature of human housekeeping genes. Trends Genet..

[6] Saxonov S., Berg P., Brutlag D.L. (2006). A genome-wide analysis of CpG dinucleotides in the human genome distinguishes two distinct classes of promoters. Proc. Natl. Acad. Sci. USA.

[7] Venter J.C., Adams M.D., Myers E.W., Li P.W., Mural R.J., Sutton G.G., Smith H.O., Yandell M., Evans C.A., Holt R.A. (2001). The sequence of the human genome. Science.

[8] Li E., Zhang Y. (2014). DNA methylation in mammals. Cold Spring Harb. Perspect. Biol..

[9] Yoder J.A., Walsh C.P., Bestor T.H. (1997). Cytosine methylation and the ecology of intragenomic parasites. Trends Genet..

[10] Hanahan D., Weinberg R.A. (2011). Hallmarks of cancer: The next generation. Cell.

[11] Hao X., Luo H., Krawczyk M., Wei W., Wang W., Wang J., Flagg K., Hou J., Zhang H., Yi S. (2017). DNA methylation markers for diagnosis and prognosis of common cancers. Proc. Natl. Acad. Sci. USA.

[12] Locke W.J., Guanzon D., Ma C., Liew Y.J., Duesing K.R., Fung K.Y.C., Ross J.P. (2019). DNA Methylation Cancer Biomarkers: Translation to the Clinic. Front. Genet..

[13] Bouras E., Karakioulaki M., Bougioukas K.I., Aivaliotis M., Tzimagiorgis G., Chourdakis M. (2019). Gene promoter methylation and cancer: An umbrella review. Gene.

[14] Leal A., Sidransky D., Brait M. (2020). Tissue and Cell-Free DNA-Based Epigenomic Approaches for Cancer Detection. Clin. Chem..

[15] Dunn B.K. (2003). Hypomethylation: One side of a larger picture. Ann. N. Y. Acad. Sci..

[16] Babaian A., Mager D.L. (2016). Endogenous retroviral promoter exaptation in human cancer. Mob. DNA.

[17] Burns K.H. (2017). Transposable elements in cancer. Nat. Rev. Cancer.

[18] Deininger P.L., Batzer M.A. (2002). Mammalian retroelements. Genome Res..

[19] Kazazian H.H. (2000). Genetics. L1 retrotransposons shape the mammalian genome. Science.

[20] Lavasanifar A., Sharp C.N., Korte E.A., Yin T., Hosseinnejad K., Jortani S.A. (2019). Long interspersed nuclear element-1 mobilization as a target in cancer diagnostics, prognostics and therapeutics. Clin. Chim. Acta.

[21] Burns K.H. (2020). Our Conflict with Transposable Elements and Its Implications for Human Disease. Annu. Rev. Pathol. Mech. Dis..

[22] Luan D.D., Korman M.H., Jakubczak J.L., Eickbush T.H. (1993). Reverse transcription of R2Bm RNA is primed by a nick at the chromosomal target site: A mechanism for non-LTR retrotransposition. Cell.

[23] Denli A.M., Narvaiza I., Kerman B.E., Pena M., Benner C., Marchetto M.C., Diedrich J.K., Aslanian A., Ma J., Moresco J.J. (2015). Primate-specific ORF0 contributes to retrotransposon-mediated diversity. Cell.

[24] Rodic N. (2018). LINE-1 activity and regulation in cancer. Front. Biosci. (Landmark Ed.).

[25] Beck C.R., Collier P., Macfarlane C., Malig M., Kidd J.M., Eichler E.E., Badge R.M., Moran J.V. (2010). LINE-1 retrotransposition activity in human genomes. Cell.

[26] Brouha B., Schustak J., Badge R.M., Lutz-Prigge S., Farley A.H., Moran J.V., Kazazian H.H. (2003). Hot L1s account for the bulk of retrotransposition in the human population. Proc. Natl. Acad. Sci. USA.

[27] Pfeifer G.P. (2018). Defining driver DNA methylation changes in human cancer. Int. J. Mol. Sci..

[28] Cajuso T., Sulo P., Tanskanen T., Katainen R., Taira A., Hänninen U.A., Kondelin J., Forsström L., Välimäki N., Aavikko M. (2019). Retrotransposon insertions can initiate colorectal cancer and are associated with poor survival. Nat. Commun..

[29] Helman E., Lawrence M.S., Stewart C., Sougnez C., Getz G., Meyerson M. (2014). Somatic retrotransposition in human cancer revealed by whole-genome and exome sequencing. Genome Res..

[30] Miki Y., Nishisho I., Horii A., Miyoshi Y., Utsunomiya J., Kinzler K.W., Vogelstein B., Nakamura Y. (1992). Disruption of the APC gene by a retrotransposal insertion of L1 sequence in a colon cancer. Cancer Res..

[31] Shukla R., Upton K.R., Muñoz-Lopez M., Gerhardt D.J., Fisher M.E., Nguyen T., Brennan P.M., Baillie J.K., Collino A., Ghisletti S. (2013). Endogenous retrotransposition activates oncogenic pathways in hepatocellular carcinoma. Cell.

[32] Robberecht C., Voet T., Zamani Esteki M., Nowakowska B.A., Vermeesch J.R. (2013). Nonallelic homologous recombination between retrotransposable elements is a driver of de novo unbalanced translocations. Genome Res..

[33] Sharif F.A. (2019). Frequency of balanced reciprocal translocations from couples with recurrent miscarriages correlates with the density of Alu and L1 repeat elements: Literature finding-based study. Middle East. J. Med. Genet..

[34] Rodriguez-Martin B., Alvarez E.G., Baez-Ortega A., Zamora J., Supek F., Demeulemeester J., Santamarina M., Ju Y.S., Temes J., Garcia-Souto D. (2020). Pan-cancer analysis of whole genomes identifies driver rearrangements promoted by LINE-1 retrotransposition. Nat. Genet..

[35] Baba Y., Murata A., Watanabe M., Baba H. (2014). Clinical implications of the LINE-1 methylation levels in patients with gastrointestinal cancer. Surg. Today.

[36] Iskow R.C., McCabe M.T., Mills R.E., Torene S., Pittard W.S., Neuwald A.F., Van Meir E.G., Vertino P.M., Devine S.E. (2010). Natural mutagenesis of human genomes by endogenous retrotransposons. Cell.

[37] Tufarelli C., Badge R.M. (2017). Retrotransposon-Driven Transcription and Cancer. Human Retrotransposons in Health and Disease.

[38] Zheng Y., Joyce B.T., Liu L., Zhang Z., Kibbe W.A., Zhang W., Hou L. (2017). Prediction of genome-wide DNA methylation in repetitive elements. Nucleic Acids Res..

[39] Hancks D.C., Kazazian H.H. (2016). Roles for retrotransposon insertions in human disease. Mob. DNA.

[40] Ishak C.A., Classon M., De Carvalho D.D. (2018). Deregulation of Retroelements as an Emerging Therapeutic Opportunity in Cancer. Trends Cancer.

[41] Tubio J.M.C., Li Y., Ju Y.S., Martincorena I., Cooke S.L., Tojo M., Gundem G., Pipinikas C.P., Zamora J., Raine K. (2014). Mobile DNA in cancer. Extensive transduction of nonrepetitive DNA mediated by L1 retrotransposition in cancer genomes. Science.

[42] Lee E., Iskow R., Yang L., Gokcumen O., Haseley P., Luquette L.J., Lohr J.G., Harris C.C., Ding L., Wilson R.K. (2012). Landscape of somatic retrotransposition in human cancers. Science.

[43] Weber B., Kimhi S., Howard G., Eden A., Lyko F. (2010). Demethylation of a LINE-1 antisense promoter in the cMet locus impairs Met signalling through induction of illegitimate transcription. Oncogene.

[44] Harrison A., Parle-McDermott A. (2011). DNA methylation: A timeline of methods and applications. Front. Genet..

[45] Kurinomaru T., Kurita R. (2017). Bisulfite-free approaches for DNA methylation profiling. Anal. Methods.

[46] Niya M.H.K., Roshan-zamir N., Mortazavi E. (2019). DNA Methylation Tools and Strategies: Methods in a Review. Asian Pac. J. Cancer Biol..

[47] Herman J.G., Graff J.R., Myöhänen S., Nelkin B.D., Baylin S.B. (1996). Methylation-specific PCR: A novel PCR assay for methylation status of CpG islands. Proc. Natl. Acad. Sci. USA.

[48] Xiong Z., Laird P.W. (1997). COBRA: A sensitive and quantitative DNA methylation assay. Nucleic Acids Res..

[49] Eads C.A., Danenberg K.D., Kawakami K., Saltz L.B., Blake C., Shibata D., Danenberg P.V., Laird P.W. (2000). MethyLight: A high-throughput assay to measure DNA methylation. Nucleic Acids Res..

[50] Radpour R., Kohler C., Haghighi M., Fan A., Holzgreve W., Zhong X. (2009). Methylation profiles of 22 candidate genes in breast cancer using high-throughput MALDI-TOF mass array. Oncogene.

[51] Rauch T., Li H., Wu X., Pfeifer G.P. (2006). MIRA-assisted microarray analysis, a new technology for the determination of DNA methylation patterns, identifies frequent methylation of homeodomain-containing genes in lung cancer cells. Cancer Res..

[52] de Maat M.F., Umetani N., Sunami E., Turner R.R., Hoon D.S. (2007). Assessment of methylation events during colorectal tumor progression by absolute quantitative analysis of methylated alleles. Mol. Cancer Res..

[53] Barchitta M., Quattrocchi A., Maugeri A., Vinciguerra M., Agodi A. (2014). LINE-1 hypomethylation in blood and tissue samples as an epigenetic marker for cancer risk: A systematic review and meta-analysis. PLoS ONE.

[54] van Hoesel A.Q., van de Velde C.J., Kuppen P.J., Liefers G.J., Putter H., Sato Y., Elashoff D.A., Turner R.R., Shamonki J.M., de Kruijf E.M. (2012). Hypomethylation of LINE-1 in primary tumor has poor prognosis in young breast cancer patients: A retrospective cohort study. Breast Cancer Res. Treat..

[55] Park S.Y., Seo A.N., Jung H.Y., Gwak J.M., Jung N., Cho N.Y., Kang G.H. (2014). Alu and LINE-1 hypomethylation is associated with HER2 enriched subtype of breast cancer. PLoS ONE.

[56] Gao X.D., Qu J.H., Chang X.J., Lu Y.Y., Bai W.L., Wang H., Xu Z.X., An L.J., Wang C.P., Zeng Z. (2014). Hypomethylation of long interspersed nuclear element-1 promoter is associated with poor outcomes for curative resected hepatocellular carcinoma. Liver Int..

[57] Zhu C., Utsunomiya T., Ikemoto T., Yamada S., Morine Y., Imura S., Arakawa Y., Takasu C., Ishikawa D., Imoto I. (2014). Hypomethylation of long interspersed nuclear element-1 (LINE-1) is associated with poor prognosis via activation of c-MET in hepatocellular carcinoma. Ann. Surg. Oncol..

[58] Anwar S.L., Hasemeier B., Schipper E., Vogel A., Kreipe H., Lehmann U. (2019). LINE-1 hypomethylation in human hepatocellular carcinomas correlates with shorter overall survival and CIMP phenotype. PLoS ONE.

[59] Shigaki H., Baba Y., Watanabe M., Iwagami S., Miyake K., Ishimoto T., Iwatsuki M., Baba H. (2012). LINE-1 hypomethylation in noncancerous esophageal mucosae is associated with smoking history. Ann. Surg. Oncol..

[60] Iwagami S., Baba Y., Watanabe M., Shigaki H., Miyake K., Ishimoto T., Iwatsuki M., Sakamaki K., Ohashi Y., Baba H. (2013). LINE-1 hypomethylation is associated with a poor prognosis among patients with curatively resected esophageal squamous cell carcinoma. Ann. Surg..

[61] Zhu J., Ling Y., Xu Y., Lu M.Z., Liu Y.P., Zhang C.S. (2015). Elevated expression of MDR1 associated with Line-1 hypomethylation in esophageal squamous cell carcinoma. Int. J. Clin. Exp. Pathol..

[62] Antelo M., Balaguer F., Shia J., Shen Y., Hur K., Moreira L., Cuatrecasas M., Bujanda L., Giraldez M.D., Takahashi M. (2012). A high degree of LINE-1 hypomethylation is a unique feature of early-onset colorectal cancer. PLoS ONE.

[63] Inamura K., Yamauchi M., Nishihara R., Lochhead P., Qian Z.R., Kuchiba A., Kim S.A., Mima K., Sukawa Y., Jung S. (2014). Tumor LINE-1 methylation level and microsatellite instability in relation to colorectal cancer prognosis. J. Natl. Cancer Inst..

[64] Kaneko M., Kotake M., Bando H., Yamada T., Takemura H., Minamoto T. (2016). Prognostic and predictive significance of long interspersed nucleotide element-1 methylation in advanced-stage colorectal cancer. BMC Cancer.

[65] Swets M., Zaalberg A., Boot A., van Wezel T., Frouws M.A., Bastiaannet E., Gelderblom H., van de Velde C.J., Kuppen P.J. (2016). Tumor LINE-1 Methylation Level in Association with Survival of Patients with Stage II Colon Cancer. Int. J. Mol. Sci..

[66] Kupcinskas J., Steponaitiene R., Langner C., Smailyte G., Skieceviciene J., Kupcinskas L., Malfertheiner P., Link A. (2017). LINE-1 hypomethylation is not a common event in preneoplastic stages of gastric carcinogenesis. Sci. Rep..

[67] Bae J.M., Shin S.H., Kwon H.J., Park S.Y., Kook M.C., Kim Y.W., Cho N.Y., Kim N., Kim T.Y., Kim D. (2012). ALU and LINE-1 hypomethylations in multistep gastric carcinogenesis and their prognostic implications. Int. J. Cancer.

[68] Ikeda K., Shiraishi K., Eguchi A., Shibata H., Yoshimoto K., Mori T., Baba Y., Baba H., Suzuki M. (2013). Long interspersed nucleotide element 1 hypomethylation is associated with poor prognosis of lung adenocarcinoma. Ann. Thorac. Surg..

[69] Rhee Y.Y., Lee T.H., Song Y.S., Wen X., Kim H., Jheon S., Lee C.T., Kim J., Cho N.Y., Chung J.H. (2015). Prognostic significance of promoter CpG island hypermethylation and repetitive DNA hypomethylation in stage I lung adenocarcinoma. Virchows Arch..

[70] Imperatori A., Sahnane N., Rotolo N., Franzi F., Nardecchia E., Libera L., Romualdi C., Cattoni M., Sessa F., Dominioni L. (2017). LINE-1 hypomethylation is associated to specific clinico-pathological features in Stage I non-small cell lung cancer. Lung Cancer.

[71] Furlan C., Polesel J., Barzan L., Franchin G., Sulfaro S., Romeo S., Colizzi F., Rizzo A., Baggio V., Giacomarra V. (2017). Prognostic significance of LINE-1 hypomethylation in oropharyngeal squamous cell carcinoma. Clin. Epigenet..

[72] Sunami E., de Maat M., Vu A., Turner R.R., Hoon D.S. (2011). LINE-1 hypomethylation during primary colon cancer progression. PLoS ONE.

[73] Corley D.A., Jensen C.D., Marks A.R., Zhao W.K., Lee J.K., Doubeni C.A., Zauber A.G., de Boer J., Fireman B.H., Schottinger J.E. (2014). Adenoma detection rate and risk of colorectal cancer and death. N. Engl. J. Med..

[74] Shah A.K., Saunders N.A., Barbour A.P., Hill M.M. (2013). Early diagnostic biomarkers for esophageal adenocarcinoma--the current state of play. Cancer Epidemiol. Biomark. Prev..

[75] Chalitchagorn K., Shuangshoti S., Hourpai N., Kongruttanachok N., Tangkijvanich P., Thong-ngam D., Voravud N., Sriuranpong V., Mutirangura A. (2004). Distinctive pattern of LINE-1 methylation level in normal tissues and the association with carcinogenesis. Oncogene.

[76] Schulz W.A., Elo J.P., Florl A.R., Pennanen S., Santourlidis S., Engers R., Buchardt M., Seifert H.H., Visakorpi T. (2002). Genomewide DNA hypomethylation is associated with alterations on chromosome 8 in prostate carcinoma. Genes Chromosomes Cancer.

[77] Santourlidis S., Florl A., Ackermann R., Wirtz H.C., Schulz W.A. (1999). High frequency of alterations in DNA methylation in adenocarcinoma of the prostate. Prostate.

[78] Pattamadilok J., Huapai N., Rattanatanyong P., Vasurattana A., Triratanachat S., Tresukosol D., Mutirangura A. (2008). LINE-1 hypomethylation level as a potential prognostic factor for epithelial ovarian cancer. Int. J. Gynecol. Cancer.

[79] Ogino S., Nosho K., Kirkner G.J., Kawasaki T., Chan A.T., Schernhammer E.S., Giovannucci E.L., Fuchs C.S. (2008). A cohort study of tumoral LINE-1 hypomethylation and prognosis in colon cancer. J. Natl. Cancer Inst..

[80] van Bemmel D., Lenz P., Liao L.M., Baris D., Sternberg L.R., Warner A., Johnson A., Jones M., Kida M., Schwenn M. (2012). Correlation of LINE-1 methylation levels in patient-matched buffy coat, serum, buccal cell, and bladder tumor tissue DNA samples. Cancer Epidemiol. Biomark. Prev..

[81] Ilie M., Hofman P. (2016). Pros: Can tissue biopsy be replaced by liquid biopsy?. Transl. Lung Cancer Res..

[82] Parikh A.R., Leshchiner I., Elagina L., Goyal L., Levovitz C., Siravegna G., Livitz D., Rhrissorrakrai K., Martin E.E., Van Seventer E.E. (2019). Liquid versus tissue biopsy for detecting acquired resistance and tumor heterogeneity in gastrointestinal cancers. Nat. Med..

[83] Cohen J.D., Li L., Wang Y., Thoburn C., Afsari B., Danilova L., Douville C., Javed A.A., Wong F., Mattox A. (2018). Detection and localization of surgically resectable cancers with a multi-analyte blood test. Science.

[84] Rothwell D.G., Ayub M., Cook N., Thistlethwaite F., Carter L., Dean E., Smith N., Villa S., Dransfield J., Clipson A. (2019). Utility of ctDNA to support patient selection for early phase clinical trials: The TARGET study. Nat. Med..

[85] Rykova E.Y., Morozkin E.S., Ponomaryova A.A., Loseva E.M., Zaporozhchenko I.A., Cherdyntseva N.V., Vlassov V.V., Laktionov P.P. (2012). Cell-free and cell-bound circulating nucleic acid complexes: Mechanisms of generation, concentration and content. Expert Opin. Biol. Ther..

[86] Warton K., Samimi G. (2015). Methylation of cell-free circulating DNA in the diagnosis of cancer. Front. Mol. Biosci..

[87] Rykova E.Y., Ponomaryova A.A., Zaporozhchenko I.A., Vlassov V.V., Cherdyntseva N.V., Laktionov P.P. (2018). Circulating DNA-based lung cancer diagnostics and follow-up: Looking for epigenetic markers. Transl. Cancer Res..

[88] Bettegowda C., Sausen M., Leary R.J., Kinde I., Wang Y., Agrawal N., Bartlett B.R., Wang H., Luber B., Alani R.M. (2014). Detection of circulating tumor DNA in early- and late-stage human malignancies. Sci. Transl. Med..

[89] Feinberg A.P., Koldobskiy M.A., Gondor A. (2016). Epigenetic modulators, modifiers and mediators in cancer aetiology and progression. Nat. Rev. Genet..

[90] Jorda M., Diez-Villanueva A., Mallona I., Martin B., Lois S., Barrera V., Esteller M., Vavouri T., Peinado M.A. (2017). The epigenetic landscape of Alu repeats delineates the structural and functional genomic architecture of colon cancer cells. Genome Res..

[91] Xiao-Jie L., Hui-Ying X., Qi X., Jiang X., Shi-Jie M. (2016). LINE-1 in cancer: Multifaceted functions and potential clinical implications. Genet. Med..

[92] Farhat F.S., Houhou W. (2013). Targeted therapies in non-small cell lung carcinoma: What have we achieved so far?. Ther. Adv. Med. Oncol..

[93] Robles A.I., Harris C.C. (2017). Integration of multiple “OMIC” biomarkers: A precision medicine strategy for lung cancer. Lung Cancer.

[94] Rizvi N.A., Hellmann M.D., Snyder A., Kvistborg P., Makarov V., Havel J.J., Lee W., Yuan J., Wong P., Ho T.S. (2015). Cancer immunology. Mutational landscape determines sensitivity to PD-1 blockade in non-small cell lung cancer. Science.

[95] El-Maarri O., Walier M., Behne F., van Uum J., Singer H., Diaz-Lacava A., Nusgen N., Niemann B., Watzka M., Reinsberg J. (2011). Methylation at global LINE-1 repeats in human blood are affected by gender but not by age or natural hormone cycles. PLoS ONE.

[96] Bollati V., Schwartz J., Wright R., Litonjua A., Tarantini L., Suh H., Sparrow D., Vokonas P., Baccarelli A. (2009). Decline in genomic DNA methylation through aging in a cohort of elderly subjects. Mech. Ageing Dev..

[97] Erichsen L., Beermann A., Arauzo-Bravo M.J., Hassan M., Dkhil M.A., Al-Quraishy S., Hafiz T.A., Fischer J.C., Santourlidis S. (2018). Genome-wide hypomethylation of LINE-1 and Alu retroelements in cell-free DNA of blood is an epigenetic biomarker of human aging. Saudi J. Biol. Sci..

[98] Terry D.M., Devine S.E. (2019). Aberrantly High Levels of Somatic LINE-1 Expression and Retrotransposition in Human Neurological Disorders. Front. Genet..

[99] Ghanjati F., Beermann A., Hermanns T., Poyet C., Arauzo-Bravo M.J., Seifert H.H., Schmidtpeter M., Goering W., Sorg R., Wernet P. (2014). Unreserved application of epigenetic methods to define differences of DNA methylation between urinary cellular and cell-free DNA. Cancer Biomark..

[100] Wolff E.M., Byun H.M., Han H.F., Sharma S., Nichols P.W., Siegmund K.D., Yang A.S., Jones P.A., Liang G. (2010). Hypomethylation of a LINE-1 promoter activates an alternate transcript of the MET oncogene in bladders with cancer. PLoS Genet..

[101] Zhong H.H., Hu S.J., Yu B., Jiang S.S., Zhang J., Luo D., Yang M.W., Su W.Y., Shao Y.L., Deng H.L. (2017). Apoptosis in the aging liver. Oncotarget.

[102] Horvath S. (2013). DNA methylation age of human tissues and cell types. Genome Biol..

[103] Wangsri S., Subbalekha K., Kitkumthorn N., Mutirangura A. (2012). Patterns and possible roles of LINE-1 methylation changes in smoke-exposed epithelia. PLoS ONE.

[104] Liu F., Killian J.K., Yang M., Walker R.L., Hong J.A., Zhang M., Davis S., Zhang Y., Hussain M., Xi S. (2010). Epigenomic alterations and gene expression profiles in respiratory epithelia exposed to cigarette smoke condensate. Oncogene.

[105] Zeidler R., Albermann K., Lang S. (2007). Nicotine and apoptosis. Apoptosis.

[106] Pox C.P., Altenhofen L., Brenner H., Theilmeier A., Von Stillfried D., Schmiegel W. (2012). Efficacy of a nationwide screening colonoscopy program for colorectal cancer. Gastroenterology.

[107] Chiu H.M., Lee Y.C., Tu C.H., Chen C.C., Tseng P.H., Liang J.T., Shun C.T., Lin J.T., Wu M.S. (2013). Association between early stage colon neoplasms and false-negative results from the fecal immunochemical test. Clin. Gastroenterol. Hepatol..

[108] Hirai H.W., Tsoi K.K., Chan J.Y., Wong S.H., Ching J.Y., Wong M.C., Wu J.C., Chan F.K., Sung J.J., Ng S.C. (2016). Systematic review with meta-analysis: Faecal occult blood tests show lower colorectal cancer detection rates in the proximal colon in colonoscopy-verified diagnostic studies. Aliment. Pharmacol. Ther..

[109] Smith R.A., Andrews K., Brooks D., DeSantis C.E., Fedewa S.A., Lortet-Tieulent J., Manassaram-Baptiste D., Brawley O.W., Wender R.C. (2016). Cancer screening in the United States, 2016: A review of current American Cancer Society guidelines and current issues in cancer screening. CA Cancer J. Clin..

[110] von Wagner C., Baio G., Raine R., Snowball J., Morris S., Atkin W., Obichere A., Handley G., Logan R.F., Rainbow S. (2011). Inequalities in participation in an organized national colorectal cancer screening programme: Results from the first 2.6 million invitations in England. Int. J. Epidemiol..

[111] Taber J.M., Aspinwall L.G., Heichman K.A., Kinney A.Y. (2014). Preferences for blood-based colon cancer screening differ by race/ethnicity. Am. J. Health Behav..

[112] Nagai Y., Sunami E., Yamamoto Y., Hata K., Okada S., Murono K., Yasuda K., Otani K., Nishikawa T., Tanaka T. (2017). LINE-1 hypomethylation status of circulating cell-free DNA in plasma as a biomarker for colorectal cancer. Oncotarget.

[113] Matsunoki A., Kawakami K., Kotake M., Kaneko M., Kitamura H., Ooi A., Watanabe G., Minamoto T. (2012). LINE-1 methylation shows little intra-patient heterogeneity in primary and synchronous metastatic colorectal cancer. BMC Cancer.

[114] Rhee Y.Y., Kim M.J., Bae J.M., Koh J.M., Cho N.Y., Juhnn Y.S., Kim D., Kang G.H. (2012). Clinical outcomes of patients with microsatellite-unstable colorectal carcinomas depend on L1 methylation level. Ann. Surg. Oncol..

[115] Gainetdinov I.V., Kapitskaya K.Y., Rykova E.Y., Ponomaryova A.A., Cherdyntseva N.V., Vlassov V.V., Laktionov P.P., Azhikina T.L. (2016). Hypomethylation of human-specific family of LINE-1 retrotransposons in circulating DNA of lung cancer patients. Lung Cancer.

[116] Ponomaryova A., Rykova E., Cherdyntseva N., Bondar A., Dobrodeev A., Zavyalov A., Tuzikov S., Bryzgalov L., Merkulova T., Vlassov V. (2016). Epigenetic probes for lung cancer monitoring: Line-1 methylation pattern in blood-circulating DNA. Russ. J. Genet. Appl. Res..

[117] Ponomaryova A.A., Cherdyntseva N.V., Bondar A.A., Dobrodeev A.Y., Zavyalov A.A., Tuzikov S.A., Vlassov V.V., Choinzonov E.L., Laktionov P.P., Rykova E.Y. (2017). Dynamics of LINE-1 Retrotransposon Methylation Levels in Circulating DNA from Lung Cancer Patients Undergoing Antitumor Therapy. Mol. Biol. (Mosk).

[118] Ponomaryova A.A., Rykova E.Y., Azhikina T.L., Bondar A.A., Cheremisina O.V., Rodionov E.O., Boyarko V.V., Laktionov P.P., Cherdyntseva N.V. (2020). Long interspersed nuclear element-1 methylation status in the circulating DNA from blood of patients with malignant and chronic inflammatory lung diseases. Eur. J. Cancer Prev..

[119] Wedge E., Hansen J.W., Garde C., Asmar F., Tholstrup D., Kristensen S.S., Munch-Petersen H.D., Ralfkiaer E., Brown P., Gronbaek K. (2017). Global hypomethylation is an independent prognostic factor in diffuse large B cell lymphoma. Am. J. Hematol..

[120] Hoshimoto S., Kuo C.T., Chong K.K., Takeshima T.L., Takei Y., Li M.W., Huang S.K., Sim M.S., Morton D.L., Hoon D.S. (2012). AIM1 and LINE-1 epigenetic aberrations in tumor and serum relate to melanoma progression and disease outcome. J. Investig. Dermatol..

[121] Gold B., Cankovic M., Furtado L.V., Meier F., Gocke C.D. (2015). Do circulating tumor cells, exosomes, and circulating tumor nucleic acids have clinical utility? A report of the association for molecular pathology. J. Mol. Diagn..

[122] Pantel K., Alix-Panabieres C. (2017). Tumour microenvironment: Informing on minimal residual disease in solid tumours. Nat. Rev. Clin. Oncol..

[123] Thierry A.R., El Messaoudi S., Gahan P.B., Anker P., Stroun M. (2016). Origins, structures, and functions of circulating DNA in oncology. Cancer Metastasis Rev..

[124] Su S.-F., de Castro Abreu A.L., Chihara Y., Tsai Y., Andreu-Vieyra C., Daneshmand S., Skinner E.C., Jones P.A., Siegmund K.D., Liang G. (2014). A panel of three markers hyper-and hypomethylated in urine sediments accurately predicts bladder cancer recurrence. Clin. Cancer Res..

[125] Kivioja T., Vähärautio A., Karlsson K., Bonke M., Enge M., Linnarsson S., Taipale J. (2012). Counting absolute numbers of molecules using unique molecular identifiers. Nat. Methods.

